# Geographic Expansion of the Invasive Mosquito *Aedes albopictus* across Panama—Implications for Control of Dengue and Chikungunya Viruses

**DOI:** 10.1371/journal.pntd.0003383

**Published:** 2015-01-08

**Authors:** Matthew J. Miller, Jose R. Loaiza

**Affiliations:** 1 Smithsonian Tropical Research Institute, Panama City, Panama; 2 Instituto de Investigaciones Científicas y Servicios de Alta Tecnología, Ciudad de Panamá, Panamá; 3 Programa Centroamericano de Maestría en Entomología, Vicerrectoría de Investigación y Postgrado, Universidad de Panamá, Ciudad de Panamá, Panamá; Australian National University, AUSTRALIA

## Background

The Asian tiger mosquito, *Aedes* (*Stegomyia*) *albopictus*, is an invasive species that has expanded its territory to over 40% of the earth’s terrestrial landmass in the last 30 years [1]. *Ae. albopictus* is an efficient vector of all serotypes of dengue, a disease that has increased in frequency over the past 30 years in the Americas [2], where it represents an annual cost of 2,100,000,000 USD per year [3]. This mosquito is also an efficient vector of the three genotypes of Chikungunya virus, a worldwide emerging pathogen that causes fever, fatigue, and joint swelling in humans. Since 2006, Chikungunya outbreaks have been increasingly recorded outside the virus’s native range in tropical Africa, perhaps because of a mutation in the virus’s envelope gene, which increases the replication and dissemination capacity of the virus in *Ae. albopictus* [4]. During the second quarter of 2014, Chikungunya has been detected throughout much of the Americas, with major outbreaks occurring in several Caribbean nations, and local transmission confirmed or suspected in the United States, Panama, Venezuela, Peru, and Chile, creating an imminent threat for humans throughout the Americas, who have no prior exposure to this infection [5].

The first cases of Chikungunya disease in Panama were reported in May 2014, occurring in nonresidents who most likely picked up the virus in their Caribbean countries of origin. On 23 July 2014, Panama’s health authority reported autochthonous transmission of Chikungunya virus. Coincidentally, the earliest cases involved patients located in Juan Diaz, an urban area on the eastern outskirts of Panama City, where the first specimen of invasive *Ae. albopictus* was collected in 2002. *Ae. albopictus* has expanded across much of Panama since that time, yet to date, no information exists about the degree of expansion or about the factors contributing to the geographic expansion of this important mosquito vector across Panama. Here, we map the temporal expansion of *Ae. albopictus*, use species distribution models to determine the ecological and nonecological factors associated with its expansion, and comment on the implications for vector and disease control programs in Panama and elsewhere in the American tropics.

## Tempo and Mode of *Ae. albopictus* Expansion in Panama

**Figure 1:**
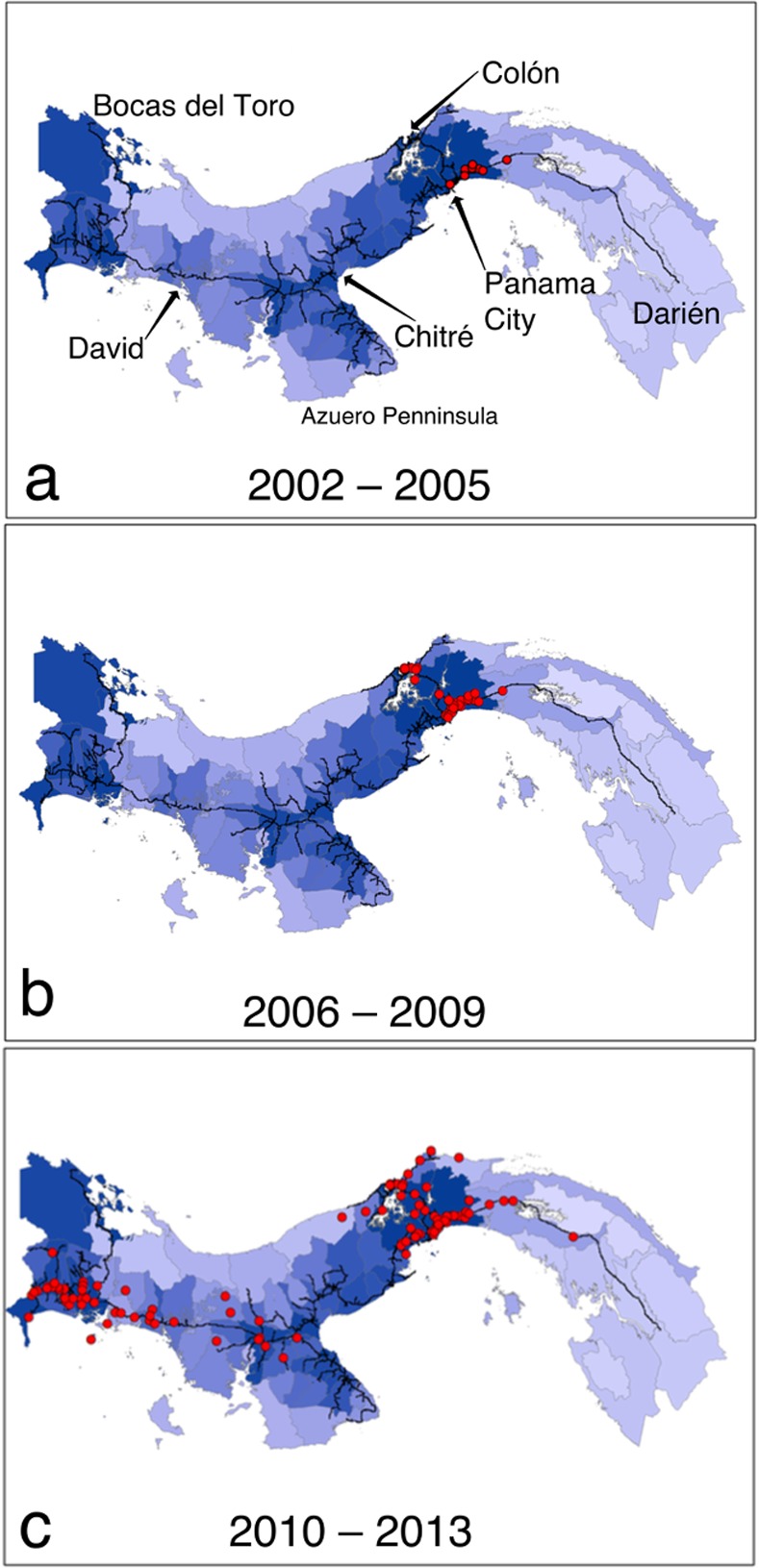
Occurrence points for *Ae. albopictus* for three time periods during its recent expansion across the Republic of Panama: A) between 2002 and 2006, *Ae. albopictus* was found only in the eastern metropolitan area of Panama City; B) during 2006 and 2009, *Ae. albopictus* expanded to the Colón on the Caribbean coast; and C) between 2010 and 2013, the species was found throughout much of western Panama as well as east of Panama City. Darker blue colors indicate political districts with higher human population densities. *Ae. albopictus* apparently has not yet spread to the Bocas del Toro province in northwestern Panama or the Azuero Peninsula, which includes the city of Chitré, nor to much of the lightly inhabited Darién province.

Panama’s Ministry of Health (MINSA) maintains a nationwide surveillance program for *Aedes* mosquitoes (S1 Methods) and provided us with geographic coordinates and dates for confirmed samples of *Ae. albopictus* collected between 2002 and 2013, which were supplemented with Jose Loaiza’s surveys of mosquitoes across Panama. Mosquito occurrence data were placed into three temporal pools: 2002–2005, 2006–2009, and 2010–2013. Between 2002 and 2005, *Ae. albopictus* was found only in the eastern portion of Panama City (Fig. 1A). Between 2006 and 2009, mosquito density increased in Panama City and also expanded to Colón, central Panama’s Caribbean port (Fig. 1B). Between 2010 and 2013, *Ae. albopictus* expanded both eastward from Panama City and also into western Panama between the Costa Rican border and Santiago, Veraguas (Fig. 1C). Although *Ae. albopictus* appears to have expanded westward from Panama City along the Pan-American highway, the lack of confirmed samples from the Azuero Peninsula east to Panama City’s western edges raise the possibility that the 2010–2013 distribution of *Ae. albopictus* in western Panama was the result of a separate colonization event from Costa Rica, as the species has occurred in several locations in that country since at least 2009 [6].

## Road Networks Alone Best Explain the Geographic Expansion of *Ae. albopictus* across Panama

We created competing species distribution models (SDMs) via maximum entropy machine learning algorithms using the Maxent software package (version 3.3) [7] to evaluate the factors associated with *Ae. albopictus* expansion. SDMs predict the suitability (i.e., probability of species occurrence, range: 0–1) of map cells based on the distribution of known occurrence points and the environmental conditions of map cells. For environmental conditions, we used all 19 WorldClim climate layers [8] as well as geographic information system (GIS) layers of principal roads and population density [9], which we rasterized and scaled to 2.5 arc minutes. SDMs were created using the 2006–2009 mosquito occurrence data, and model fit was evaluated by comparing the later (2010–2013) occurrence data against the model-predicted suitability of those points. We created seven SDMs including “only climate,” “only human density,” and “only roads,” as well as all possible combinations of those three datasets. All SDMs were generated based on ten replicates using cross validation and 10,000 background points, and we set our threshold for habitat suitability at 10% of all observed occurrences. We used the 2010–2013 sample data to compare the fit of each SDM in two ways: first, we calculated the mean modeled suitability of all 2010–2013 occurrence points, and second, we calculated the percentage of those occurrence points having a predicted suitability above the 10% minimum suitability threshold.

An SDM based only on the road network best predicted the 2010–2013 distribution of *Ae. albopictus* in Panama, compared to SDMs based on climate or human population density or even to models that included roads and other factors (Table 1; Fig. 1). The average suitability of 2010–2013 occurrences based on roads alone was 0.487, compared to average suitability of other models that ranged from 0.285–0.319. Likewise, 80% of the 2010–2013 samples occurred in areas predicted to be suitable habitats for *Ae. albopictus* in the roads-only model, compared to frequencies ranging from 34%–53% in other models. Interestingly, climate alone was the poorest predictor of suitability, predicting habitat suitability for only 34% of the points at which *Ae. albopictus* was actually sampled between 2010–2013 (Table 1). Our findings appear to be unbiased by mosquito sampling effort or by the relative intensity of sampling along versus off the principal road network (S1 Fig.).

**Table 1:** Performance of various geographic species distribution models to predict the expansion of *Ae. albopictus* in Panama.

**Model**	**Area under the ROC Curve (AUC)**	**Average Suitability of 2010–2013 Occurrence Points**	**2006–2009 10% Occurrence Threshold**	**% of 2010–2013 Occurrence Points above Threshold**
Roads Only	0.881	0.487	0.600	80%
Population Density Only	0.957	0.302	0.239	53%
Roads and Population Density	0.975	0.319	0.329	49%
Climate, Roads, and Population Density	0.986	0.291	0.369	39%
Roads and Climate	0.894	0.306	0.353	36%
Climate and Population Density	0.982	0.285	0.383	35%
Climate Only	0.979	0.309	0.438	34%

Models were parameterized using occurrence points sampled between 2006 and 2009. Area under the curve (AUC) measures the efficiency of the model to discriminate occurrences from random background points; AUC ranks did not correlate with model predictive performance. Model performance was evaluated using two criteria based on 2010–2013 occurrence points: first, by averaging the predicted suitability of all 110 occurrence points, and second, by calculating the frequency of those occurrence points having a predicted suitability above the 10% model threshold.

## Global versus Local Scales of *Ae. albopictus* Expansion

In general, our results agree with the global pattern of rapid expansion for *Ae. albopictus*, which is mainly attributed to human-aided dispersal [1]. Earlier studies have accurately predicted the global expansion of *Ae. albopictus* using climate-based SDMs [10,11]. Our results should not be seen as in conflict with those findings; rather, they demonstrate the dynamics of *Aedes* invasions on differing scales of time and space. At global scales, all of Panama is within the climate threshold for *Ae. albopictus* [10,11]; therefore, the immediate geographic spread across Panama is likely to be determined by factors other than ecology. Likewise, international expansion of *Ae. albopictus* has occurred principally via oceanic container vessels and/or international air traffic [1], yet our results confirm the primacy of road networks for determining patterns of *Aedes* expansion and distributional limits at local scales [12,13].

## Interactions with *Ae. aegypti* and Implications for Dengue and Chikungunya Control

Panama’s current urban mosquito control programs focus primarily on *Ae. aegypti*, yet both this species and *Ae. albopictus* are vectors of Chikungunya and dengue viruses [4,5]. Some evidence from outside the Americas suggests that reducing *Ae. aegypti* populations may be less effective at reducing Chikungunya and dengue outbreaks in Panama if simultaneous efforts to reduce the population of *Ae. albopictus* are not undertaken [14,15]. At the same time, these efforts might facilitate the ecological replacement of *Ae. aegypti* by *Ae. albopictus*, which could have both favorable and unfavorable consequences that are difficult to predict a priori. For example, there is evidence that *Ae. aegypti* is a more efficient vector of dengue virus than *Ae. albopictus* [16], which may be the result of a greater preference for human bite targets among *Ae. aegypti* than among *Ae. albopictus* [17]. Additionally, the particular strain of Chikungunya virus currently circulating in the Americas lacks the mutation allowing for selectively enhanced transmission efficiency in *Ae. albopictus* [5]. On the other hand, current vector control programs include the indoor application of insecticide in urban areas of Panama, taking advantage of the fact that *Ae. aegypti* tends to rest inside dwellings rather than in vegetation outside homes [18], while the latter is the preferred resting habitat of *Ae. albopictus* [17]. However, *Ae. albopictus* may be ecologically more plastic than *Ae. aegypti* [17], and it is likely only a matter of time until the mutations favoring Chikungunya transmission in *Ae. albopictus* migrate to the Americas.

**Figure 2:**
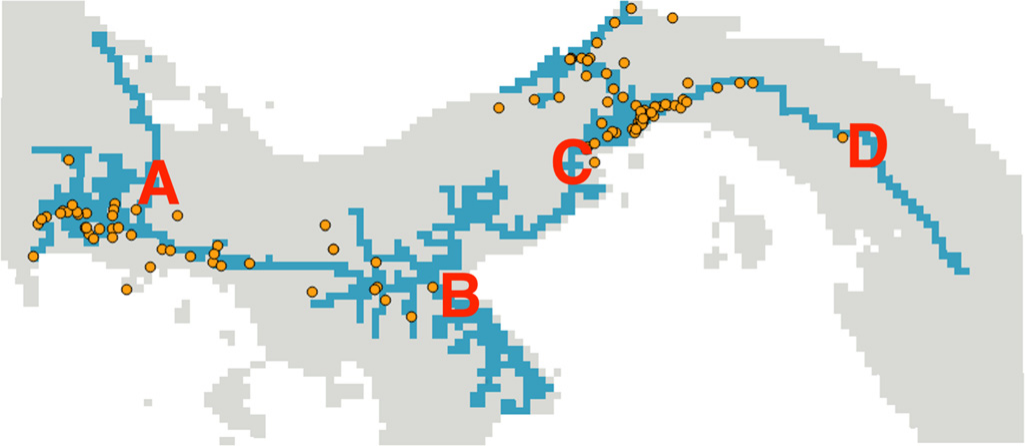
Geographic model predicting future range expansion of *Ae. albopictus* in Panama. This model is based on the best-performing-species distribution model (highway network model). Blue pixels represent locations predicted to be likely areas of *Ae. albopictus* expansion, whereas gray pixels represent areas that had a model suitability that was below the minimum threshold and therefore were unlikely to harbor mosquitoes. Orange points represent species occurrences sampled between 2010 and 2013. A series of surveillance and fumigation chokepoints at strategic locations on the highway network (e.g., points A, B, C, and D) could limit the continued expansion of *Ae. albopictus* as a first step to reduce the epidemiological risk posed by this invasive vector.

Our model presents implications for the control of dengue and Chikungunya disease. The road-only model predicts future expansion of *Ae. albopictus* into northwestern and eastern Panama as well as in the Azuero Peninsula, which includes Chitré, Panama’s third largest urban area (Fig. 2). This presents an immediate opportunity for Panama’s Ministry of Health to control the expansion of *Ae. albopictus*. Evidence from Europe suggests that passive transport of larvae occurs in items in which open water accumulates, such as used tires, while adults can be passively transported inside the cabin of cars and trucks [19]. Specifically, we recommend the fumigation of vehicles at transportation checkpoints (see suggested checkpoints in Fig. 2), which could stop the movement of adults and immature stages of *Ae. albopictus* across Panama.

Finally, our results present a cautionary tale in the face of proposals to release genetically modified *Ae. aegypti* (GM programs); trial GM program releases began in Panama in May 2014. Given that *Ae. aegypti* has similar demographic and dispersal patterns as *Ae. albopictus* [13], *Ae. aegypti* populations may quickly rebound via recolonization after cessation of GM programs. Thus, GM strategies might have only short-term effects on vector population size and may commit Panama to a repeated and costly program for long-term arbovirus control [20]. Additionally such programs could increase the chance that *Ae. albopictus* displaces *Ae. aegypti*, making the GM program less relevant. We encourage health authorities in Panama and elsewhere in tropical America to fully consider the ecology of *Ae. albopictus* alongside *Ae. aegypti* when developing dengue and Chikungunya disease control programs.

## Supporting Information

S1 Fig A map of 2010–2013 *Ae. albopictus* occurrences (yellow points) compared to Ministry of Health (MINSA) occurrence points for *Ae. aegypti* (gray points). MINSA surveys exhaustively across Panama for mosquitoes of medical importance, recording positive species occurrences, but they do not tabulate negative samples. In order to estimate sampling intensity and the proportion of sampling effort along the principle road network (gray lines), we plotted *Ae. aegypti* data that were provided to us by MINSA for the years 2007–2010. These points serve as a proxy for MINSA sampling effort. Comparing these points to the 2010–2013 *Ae. albopictus* occurrences and the road network demonstrates that MINSA intensively sampled for mosquitoes in areas such as Bocas del Toro and the eastern Azuero Peninsula where *Ae. albopictus* was not recorded and also routinely sampled in areas such as much of eastern Panama where no roads occur.
(PDF)

S1 Methods Sampling strategies for adult and immature stages of *Aedes* mosquitoes and other medically important mosquito species in Panama.
(DOCX)
