# Supplementary figures and images for: Geographic Expansion of the Invasive Mosquito Aedes albopictus across Panama—Implications for Control of Dengue and Chikungunya Viruses
Source: PLoS Negl Trop Dis. 2015 Jan 8;9(1):e0003383. doi: 10.1371/journal.pntd.0003383 (PMC4287627; doi:10.1371/journal.pntd.0003383)

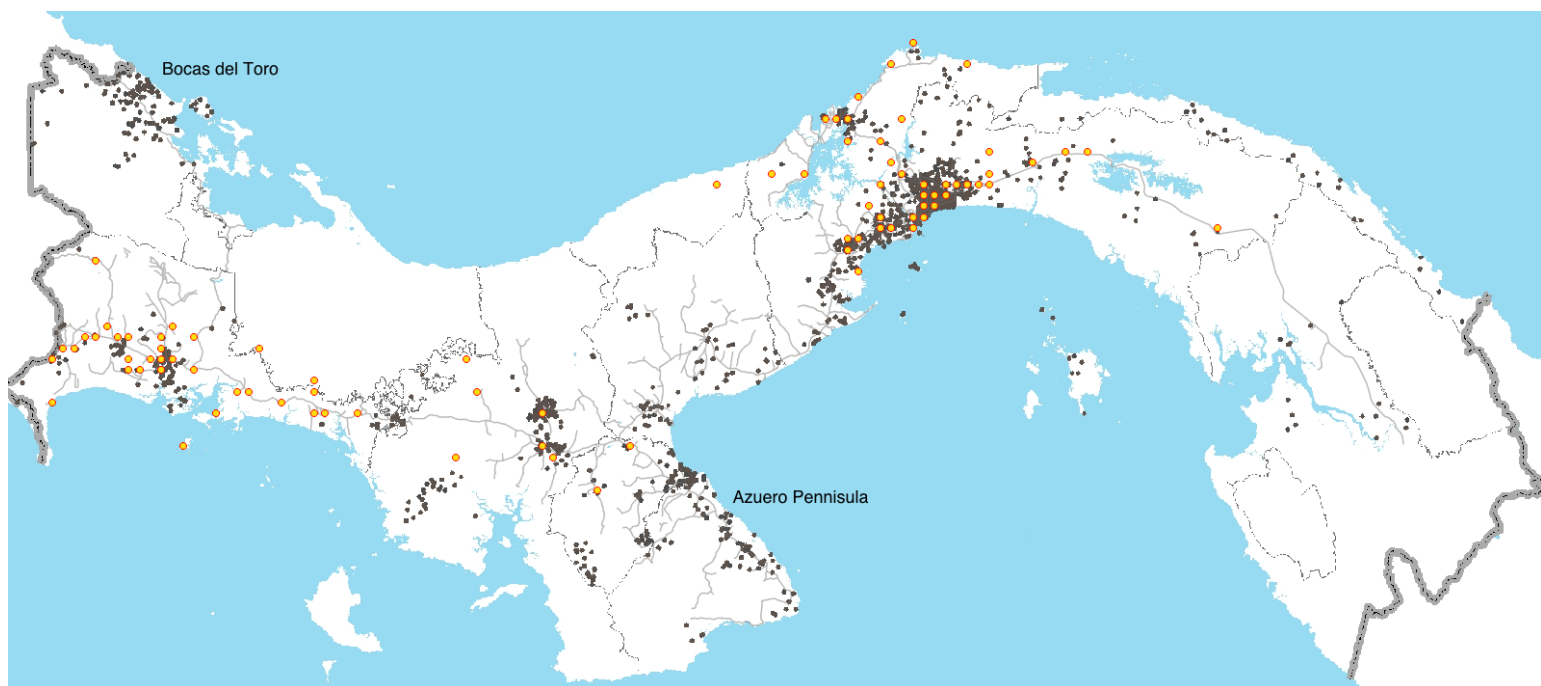

Supplement: S1 Fig — MINSA surveys exhaustively across Panama for mosquitoes of medical importance, recording positive species occurrences, but they do not tabulate negative samples. In order to estimate sampling intensity and the proportion of sampling effort along the principle road network (gray lines), we plotted Ae. aegypti data that were provided to us by MINSA for the years 2007–2010. These points serve as a proxy for MINSA sampling effort. Comparing these points to the 2010–2013 Ae. albopictus occurrences and the road network demonstrates that MINSA intensively sampled for mosquitoes in areas such as Bocas del Toro and the eastern Azuero Peninsula where Ae. albopictus was not recorded and also routinely sampled in areas such as much of eastern Panama where no roads occur. (PDF) [file pntd.0003383.s001.pdf]
