# Supplementary material for: Geographic Expansion of the Invasive Mosquito Aedes albopictus across Panama—Implications for Control of Dengue and Chikungunya Viruses
Source: PLoS Negl Trop Dis. 2015 Jan 8;9(1):e0003383. doi: 10.1371/journal.pntd.0003383 (PMC4287627; doi:10.1371/journal.pntd.0003383)
Supplement: S1 Methods — Sampling strategies for adult and immature stages of Aedes mosquitoes and other medically important mosquito species in Panama. (DOCX) [file pntd.0003383.s002.docx]

Methods S1: Supplementary Methods.

Entomology personnel at MINSA’s vector control department surveys for both adult and immature stages of *Aedes* mosquitoes (as well as other medically important mosquito species) across Panama using several methods. Adult mosquitoes are collected either resting inside houses and on the surrounding vegetation or through human landing catches using manual aspirators. Eggs, larvae and pupae are sampled using standard oviposition traps as well as via active searches of both indoor and outdoor in natural and artificial water containers. Surveys are undertaken in both urban and rural communities, including indigenous *comarcas*. In populated areas, typically, 10 to 15% of all the houses in a given neighborhood (*manzanas*) are randomly selected for mosquito surveillance, which is conducted every other month during the entire year. Information on the relative abundance of *Aedes* mosquito species is recorded, tabulated and analyzed to target places for vector control activities. Identification of mosquitoes is carried out by medical entomologists at MINSA using morphological keys, and when necessary confirmed by DNA barcoding at NAOS marine laboratory of the Smithsonian Tropical Research Institute.
